# FIXATION METHODS IN LATARJET: BIOMECHANICAL COMPARISON OF SCREW TYPES AND PLATE FIXATION

**DOI:** 10.1590/1413-785220233102e260966

**Published:** 2023-06-09

**Authors:** UFUK ARZU, MEHMET ERSIN, MEHMET CHODZA, KORAY ŞAHIN, ÖNDER KILIÇOĞLU, ALI ERŞEN

**Affiliations:** 1. VKV American Hospital, Department of Orthopaedics and Traumatology, Istanbul, Turkey.; 2. Haseki Education and Research Hospital, Department of Orthopaedics and Traumatology, Istanbul, Turkey.; 3. Bezmialem Vakıf University, Department of Orthopaedics and Traumatology, Istanbul, Turkey.; 4. Koc University, Department of Orthopaedics and Traumatology, Istanbul, Turkey.; 5. Istanbul Faculty of Medicine, Istanbul, Turkey.

**Keywords:** Shoulder joint, Surgical procedures, operative, Biomechanical phenomena, Articulação glenoumeral, Procedimentos cirúrgicos operatórios, Fenômenos biomecânicos

## Abstract

**Objective:**

Latarjet procedure is often preferred in recurrent shoulder dislocations accompanied by glenoid bone loss. It is observed that the superiority of bone graft fixation methods is still controversial. The aim of this study is to biomechanically compare the bone graft fixation methods in the Latarjet procedure.

**Methods:**

15 third-generation scapula bone models were divided into 3 groups. Graft was fixated in the first group with fully-threaded cortical screws of 3.5mm diameter, in the second group two 16 mm partially-threaded cannulated screws of 4.5mm diameter, and in the third group via a mini plate and screw. The hemispherical humeral head was placed on the tip of the cyclic charge device, and thus, the charge applied to the coracoid graft was homogeneous.

**Results:**

No statistically significant difference was found between paired comparisons (p>0.05). The forces in 5 mm displacement in total vary between 502-857N. Total stiffness measurements ranged between 105 and 625; the mean value was 258.13±53.54 with no statistically significant difference by groups (p = 0.958).

**Conclusion:**

This biomechanical study showed that there is no difference between three coracoid fixation options in terms of fixation strength. Unlike previous assumptions, plate fixation is not biomechanically superior to screw fixation. Surgeons should consider their personal preferences and experience in choosing fixation methods.

## RESUMO

**Objetivo:**

O procedimento Latarjet é normalmente preferencial em deslocamentos recorrentes do ombro acompanhados por perda óssea da glenóide. Observa-se que a superioridade dos métodos de fixação dos enxertos ósseos ainda é controversa. O objetivo deste estudo é comparar biomecanicamente os métodos de fixação de enxerto ósseo no procedimento Latarjet.

**Métodos:**

15 modelos de escápulas de terceira geração foram divididos em 3 grupos. O enxerto foi fixado no primeiro grupo com parafusos corticais totalmente rosqueados com 3,5 mm de diâmetro, no segundo grupo com dois parafusos canulados parcialmente rosqueados de 16 mm de diâmetro de 4,5 mm e no terceiro grupo através de miniplaca e parafuso. A cabeça hemisférica umeral foi colocada na ponta do dispositivo de carga cíclica e, desta forma, a carga aplicada ao enxerto coracoide foi homogênea.

**Resultados:**

Nenhuma diferença estatisticamente significativa foi encontrada entre as comparações pareadas (p>0,05). As forças em 5 mm de deslocamento no total variam entre 502-857N. As medidas de rigidez total variaram entre 105 e 625 e o valor médio foi 258,13±53,54, sem diferença estatisticamente significativa por grupos (p = 0,958).

**Conclusão:**

Este estudo biomecânico mostrou que não há diferença entre três opções de fixação de coracoides em termos de resistência à fixação. Ao contrário de suposições anteriores, a fixação de placas não é biomecanicamente superior à fixação de parafusos. Os cirurgiões devem considerar suas preferências pessoais e sua experiência na escolha de métodos de fixação.

## INTRODUCTION

Capsulolabral repair procedures, so called Bankart repair, and coracoid bone block transfer procedures are two options for surgical management of anterior shoulder instability that are currently used in clinical practice.^1^ However; in the setting of recurrent anterior shoulder instability, caution should be paid to osseous structure of the glenoid as it is an important factor for clinical outcomes.^2 , 3^ Isolated capsulolabral repair in management of recurrent shoulder instability with significant glenoid bone loss is associated with high recurrence rates.^4 , 5^ Presence of bony defects on glenoid and humeral head or insufficiency of soft tissue which is required to perform a capsulolabral repair are main indications for coracoid bone block transfer procedures which have become the gold standard for treatment of recurrent anterior shoulder instability with various techniques described.^6^

Latarjet procedure has been used with an increasing frequency and has become the gold standard treatment for treatment of recurrent shoulder instability with glenoid bone defect since its first description in 1954.^7^ This procedure consists of transfer of coracoid process, like Bristow procedure, along with conjoined tendon through a split of subscapularis muscle to anteroinferior portion of the glenoid^8 , 9^ and has been demonstrated to be very successful in treatment of shoulder instability in young athletes with bony defects or in patients with hyperlaxity.^10 , 11^

Stable and strong initial fixation of transferred bone block is a prerequisite for success of this procedure in order to minimize the risk of non-union and to initiate a reliable rehabilitation with early mobilization. Non-union was reported to be one of the most common causes of recurrence and it has been shown that failure mechanism was triggered by non-union in 42.3% and by graft resorption in 23.1% of the cases.^12 , 13^ Despite multiple modifications; rationale of this procedure mostly remains the same. Fixation of the coracoid process most commonly performed using two parallel screws with good long-term outcomes and high fusion rates.^14 , 15^ Different implants for fixation have also been proposed such as interference screws^15^ or plates.^16^ Many previous studies comparing different fixation methods have been conducted as the importance of the initial stability and strength of coracoid fixation was understood.^15 , 17 - 19^ Fixation of coracoid graft using mini-plate has been favored by some authors with consideration of stronger initial fixation, better stability, uniform load distribution between graft and glenoid and therefore faster union.^20^ However; to our knowledge, there are no biomechanical study comparing fixation strength of mini-plate to other fixation implants in current literature.

The purpose of this biomechanical study was to compare strength of initial fixation, load-to-failure and mode of failure of Latarjet procedure performed by two cortical screws, two partially-threaded cannulated screws and mini-plate. We hypothesized that there would be no significant biomechanical difference among three fixation methods.

## METHODS

This study was approved by the institutional review board and was performed in accordance with the principles of the Declaration of Helsinki. The ethics committee protocol approval number is 2017-475636. Fifteen third-generation scapula bone models (Selbone®) were obtained. A bony defect which covers 25% of the articular surface of glenoid on anteroinferior portion was created by cutting saws using a template adapted to glenoid surface of each bone model similarly as described by Itoi et al.^21^ Afterwards, each bone model underwent coracoid transfer procedure according to the technique described by Latarjet.^7^ Coracoid osteotomy was performed using a 10x0.5 mm cutting saw at coracoid base with graft length 20-25 mm. The concave inferior surface of the coracoid process was rasped and flattened in order to obtain a better fixation into the defective region of glenoid.

Fifteen samples were allocated into 3 groups. Group 1 consisted of fixation using two 3.5mm fully threaded cortical screws (Response Ortho NJ, USA). In second group, fixations were performed using two 16 mm partially threaded cannulated screws with 4.5mm diameter (Response Ortho NJ, USA) and in third group, mini plate and screws (Arthrex®, USA) were used for fixation.

**Figure 1:**
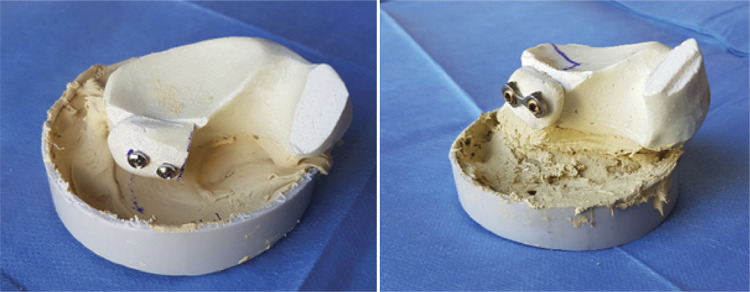
Sample of 2 cortical screw fixation and mini-plate fixation.

In group 1, following the creation of glenoid defect and coracoid osteotomy, two holes were drilled through coracoid graft using a 3.5mm cannulated drill bit over two parallel Kirschner wires. Then, the coracoid graft was positioned onto defective glenoid area flush with or slightly embedded (<1mm) to articular surface and two glenoid holes were drilled through two holes which were drilled on coracoid graft using a 2.5mm drill bit while holding the graft in correct position. Fixation was then performed using two 3.5 mm fully threaded cortical screws at appropriate length following length measurements and specimens were prepared for tests. ( Figure 1 )

In group 2, coracoid graft was positioned in a similar way to group 1 and temporarily fixed to glenoid using two parallel Kirschner wires. Then both coracoid graft and glenoid holes were drilled using a 3.2mm cannulated drill bit over these K-wires. Following length measurements, fixation was performed using two 16 mm partially threaded cannulated screws of 4.5mm diameter. After ensuring that the threads of cannulated screws are attached to the glenoid neck, which is the most distal cortex, in order to avoid rotational displacement, K-wires were removed and specimens were prepared for tests. ( Figure 1 )

In group 3, osteotomized coracoid graft was positioned to the defective area similarly to group 1 and group 2 and temporarily fixed using two parallel K-wires paying attention to plate hole positions. Then, both graft and glenoid holes were drilled using a 3.2mm cannulated drill bit. Following length measurements, plate was inserted over K-wires and fixation was performed using two 4.5 mm partially-threaded cannulated screws. Attention was paid that notches of the plate were in contact with graft cortex and compressed the coracoid. ( Figure 1 )

Each specimen was inserted into a round-shaped, polyvinyl chloride container filled with polyester paste and benzoyl peroxide, which is a hardener and accelerator, and held in adequate position until the specimen is solidified. ( Figure 1 )

### Biomechanical Test

**Figure 2:**
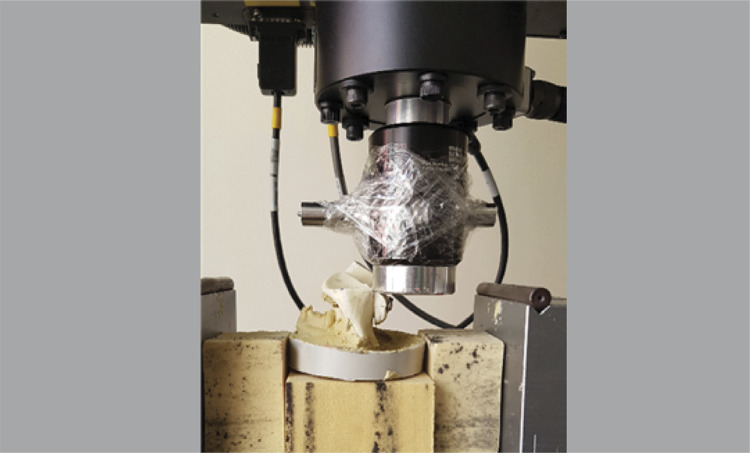
Simulate the worst-case scenario.

All samples were subject to testing with an electrodynamics test device (MTS Acumen ™ Electrodynamic Test Systems, Eden Prairie, MN, USA). The test protocol has been prepared according to previous biomechanical studies.^17 , 18^ The hemispherical stainless-steel part that would simulate the humeral head was placed on the tip of cyclic charge device thus, homogeneous load distribution to graft was aimed. The prepared samples were inserted to test device so that a vertical load to coracoid graft would be applied in order to simulate the worst-case scenario. ( Figure 2 )

**Figure 3:**
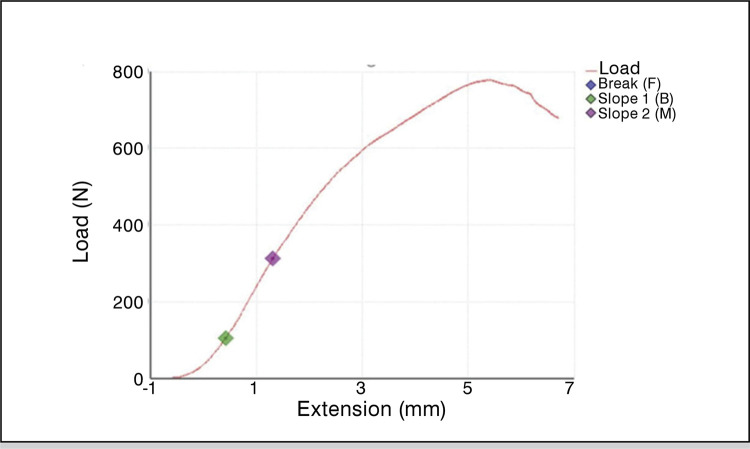
Sample of load-displacement graph.

In order to precondition the construct, 100 cycles of load were performed between 0 and 20N and a break of 30 minutes was carried out following preconditioning. Then the constructs were tested to failure with static load applied to all specimens with loading speed set to 1 mm/minute until macroscopic failure occurs. A load-displacement graph ( Figure 3 ) was obtained for each tested specimen and data obtained from the test device. On the basis of previous data by Giles et al.^22^ failure was determined as 5mm displacement of the graft relative to its initial position. The primary outcome was determined as load required in Newton (N) for 5mm of graft displacement (load-to-failure). Load-to-failure results and mode of failure were documented for data analysis.

### Statistical Analysis

All analyses were performed using GraphPad Prism Software for Windows (Version 8.0.1, San Diego, California, USA). Mean, standard deviation, median, range, minimum and maximum were used as descriptive statistical methods in order to analyse the data. Distribution of variables was tested using Shapiro-Wilk test and Kolmogorov-Smirnov test. Comparisons between three study groups were performed using analysis of variance (ANOVA). Post-hoc pairwise comparisons between groups were performed using Tukey’s. The significance level was at p=0.05 for all analyses. A post-hoc power analysis was performed on to primary outcome (load-to-failure) using (G*Power software version 3.1.9.6; Germany).

## RESULTS

**Figure 4:**
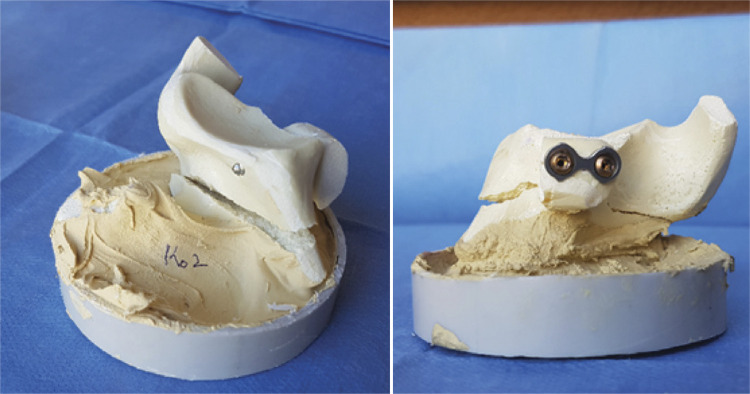
Glenoid neck fracture during the test.

The mode of failure in group 1 was complete screw pull-out without screw deformation in four cases (80%) and glenoid fracture in one case (20%). In group 2, mode of failure was screw pull-out in three cases (60%) and glenoid fracture in two cases (40%). The mode of failure with plate fixation was screw pull-out in two cases (40%) and glenoid fracture in three cases (60%) ( Figure 4 ). None of the samples failed between container-bone model interface.

**Figure 5:**
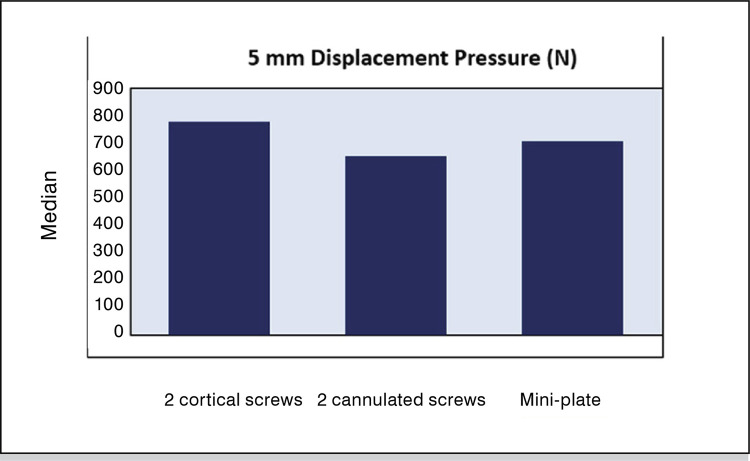
Difference between three groups.

Overall load-to-failure ranged between 502-857 N with a mean of 700±109N. Mean load-to-failure was 707.8±116.9 N (range: 545-800N), 687.8±99.3N (range: 587-810N) and 705.2±132.1N (range: 502-857N) respectively in three study groups. No statistically significant difference was observed between three groups (p=0.958). ( Figure 5 ), ( Table 1 )

**Table 1:** Load-to-failure values of three study groups.

	Load-to-failure (N)		
	n	Min-Max (Median)	Mean±SD
Group 1 (cortical screw)	5	545-800 (779)	707.8 ± 116.9
Group 2 (cannulated screw)	5	587-810 (654)	687.8 ± 99.3
Group 3 (plate)	5	502-857 (706)	705.2 ± 132.1
^b^p(Group 1 vs Group 2)		0.961	
^b^p(Group 1 vs Group 3)		0.999	
^b^p(Group 2 vs Group 3)		0.970	
Total ^a^p=0.958	15	502-857 (638)	700.26 ± 248.75

(Min: minimum, Max: maximum, SD: standard deviation, ^a^: one-way ANOVA test, ^b^: Tukey’s test).

Two-tailed post-hoc power analysis revealed that the power of the study was 81.9% for load-to-failure with 15 samples and α=٠.٠٥.

## DISCUSSION

The main finding of the present study was that accordingly to our hypothesis, initial strength and stability of the coracoid fixation did not differ biomechanically between cortical screws, cannulated screws and mini-plate. These findings implicate that surgeons may choose the fixation methods based on their experience and preference without significantly altering the construct biomechanically.

The Latarjet procedure is being more frequently used in recent years and successful results have been reported in management of recurrent shoulder instability.^23 - 25^ However; complication rate following bone block transfer procedures has been reported to be between 15 and 30%.^6 , 26^ Reported complications include infection, nerve injuries, glenoid fracture, graft non-union or osteolysis and recurrent instability.^6 , 27 - 31^ Accurate positioning and proper fixation of the coracoid graft have been reported to be essential for clinical success of this procedure in order to withstand the axial and shear forces of the glenoid joint and to avoid fixation failure which can lead to graft non-union and recurrent instability.^5 , 26^ Therefore, choosing the optimal fixation method plays an important role for success of this procedure.

Screw fixation is the most commonly used technique for fixation of coracoid graft and studied by many previous biomechanical or clinical studies. In their cadaveric study, Shin et al. did not find significant biomechanical difference between different screw types (cancellous, cortical and cannulated screws) and fixation methods (unicortical and bicortical).^18^ Another cadaveric study by Weppe et al. compared the initial fixation strength of two metal bicortical screws and a bioabsorbable interference screw and showed that metal screws provided stronger fixation.^15^ A recent biomechanical study by Alvi et al. biomechanically compared solid and cannulated screws and consistently found no significant difference in terms of load or cycles to failure.^19^

Anatomical proximity of the suprascapular nerve to exit sites of the screws in bicortical fixation method poses the possibility of an iatrogenic injury of the suprascapular nerve due to drilling or to the prominence of the screws.^32 , 33^ Therefore, fixation with two unicortical screws has been proposed in order to avoid possible nerve injury. However; in contrast to previous study by Shin et al.^18^ Schmiddem et al. recently showed that monocortical fixation was significantly weaker compared to bicortical fixation.^17^ In our study, we performed bicortical fixation, which is a prerequisite in order to obtain sufficient initial fixation strength to our opinion and consistently to previous data, we found no biomechanical difference between cortical and cannulated screws.

The Latarjet mini-plate has been developed in order to obtain better biomechanical properties and is thought to enhance compression of the graft to glenoid bone surface. The plate has a wedge profile and allows medial rotation of the coracoid graft with applied compression and therefore improves the contact between coracoid graft and glenoid bone surface. The figure of eight^8^ configuration of the plate provides a better torsional orientation and four spikes of the plate improves the stability in plate-graft interface. Consequently, these properties are believed to allow even distribution of the load to the bone compared to conventional screw fixation methods. A retrospective case series by Chaudhary et al. reported outcomes of 24 patients with failed arthroscopic Bankart repair and who were treated with Latarjet procedure using mini-plate fixation. Authors reported good clinical outcomes and no recurrence of instability.^20^ Another study by Di Giacomo et al. clinically and radiologically compared their results with mini-plate fixation to the results of their previous study without using mini-plate. They divided the coracoid bone graft into eight parts and evaluated for osteolysis using post-operative computed tomography scans. Their results showed that only deep part of the distal coracoid was significantly less involved in osteolysis with mini-plate fixation without any clinical difference. Authors concluded that mini-plate fixation did not provide reduced risk for graft osteolysis but they recommended its use to improve graft stability.^16^ However; there is a paucity of data concerning biomechanical properties of mini-plate fixation in Latarjet procedure and to our knowledge, our study is the first biomechanical study comparing mini-plate fixation to different screw fixation methods. The findings of our study revealed that mini-plate fixation was biomechanically comparable to fixation with two cortical or cannulated screws.

There are several limitations to the present study which are inherent to a biomechanical bone model study. Inability of the biomechnical test setup to reflect in vivo mechanics may be listed as a limitation of our study. Eventhough our test setup aimed to assess strength of graft fixation rather than glenohumeral joint stability, effects of soft tissue structures such as sling effect of conjoined tendon, capsular repair and subscapularis muscle could not be represented in a biomechanical test setup with bone models. Another limitation is that all native forces effecting coracoid graft, such as counter pull force produced by biceps muscle which is a contributing factor for fixation failure especially in early post-operative period, could not be reproduced in our test model. The load was applied in a single direction, perpendicular to coracoid graft which may not accurately represent in vivo graft loading after Latarjet procedure. Also due to our test mechanism we have been tested only load-to-failure loading. Absence of cyclical loading, which is known as another failure mechanism of Latarjet procedure, is another limitation of our study. However, we simulated the worst-case scenario which allowed better standardization of the load magnitude acting on the graft. Finally, the study was conducted as time-zero analysis therefore post-union biomechanics could not be evaluated.

There are also some strengths of the study. Due to homogeneity of the used bone models, possible standardization problems related to cadavers (age, gender, bone quality) have been avoided. A uniform surgical technique was performed and biomechanical testing was designed and standardized to simulate the worst-case scenario.

## CONCLUSION

Strength of initial fixation is essential for the success of Latarjet procedure and the findings of this biomechanical study showed that no difference exists between three coracoid fixation options (cortical screws, cannulated screws and mini plate) in terms of fixation strength. Unlike previous assumptions, plate fixation is not biomechanically superior to screw fixation. Surgeons should consider their personal preference and experience choosing fixation methods but further research with high evidence are needed.
